# Evaluation of the Retinal Vessel Density and Subfoveal Choroidal Thickness in Central Cerous Chorioretinopathy Using Optical Coherence Tomography

**DOI:** 10.7759/cureus.38691

**Published:** 2023-05-08

**Authors:** Thanh K Doan, Vy N T Trinh, Thanh Van Phan-Nguyen, Chuc H Nguyen

**Affiliations:** 1 Ophthalmology, Pham Ngoc Thach University of Medicine, Ho Chi Minh City, VNM; 2 Biochemistry, Pham Ngoc Thach University of Medicine, Ho Chi Minh City, VNM

**Keywords:** superficial retinal vessel density, subfoveal choroidal thickness, oct a, sd-oct, central serous chorioretinopathy

## Abstract

Purpose: The purpose of this study was to quantitatively assess changes in the subfoveal choroidal thickness (SFCT) and superficial retinal vessel density (SRVD) in acute and chronic central serous chorioretinopathy (CSCR) patients, and estimate the correlation of SFCT and SRVD with best-corrected visual acuity (BCVA), respectively, using spectral domain optical coherence tomography (SD-OCT) and optical coherence tomography angiography (OCTA).

Methods: This was a cross-sectional, case-control study. The study included CSCR patients treated at the Ho Chi Minh City Eye Hospital from May 2022 to October 2022.

Results: A total of 91 subjects (182 eyes) were included in this study, with 74 eyes in the unilateral acute CSCR group and 17 eyes in the unilateral chronic CSCR group; 91 eyes in the control group were patients' unaffected other eyes. The mean age was 40.78 ± 1.26 years (ranging from 31 to 45 years). The proportions of male and female patients were 78.0% and 22.0%, respectively. The major symptom was reduced vision, and the mean BCVA was 0.36 ± 0.05 logMAR. The mean SFCT of CSCR eyes was 357.2 ± 11.8 μm, which was 290.4 ± 8.5 μm in the control group (p < 0.05). The mean SRVD of chronic CSCR (24.2 ± 4.94%) and acute CSCR (28 ± 2.33%) eyes was lower compared with the control group (21.7 ± 1.87%). SFCT had a correlation with BCVA (r = -0.490, p < 0.05) in chronic CSCR; the center region of SRVD was likewise correlated with BCVA (r = -0.384, p < 0.05) and the parafoveal region of SRVD was also correlated with BCVA (r = -0.271, p < 0.05).

Conclusion: Both altered SFCT and SRVD were identified in CSCR patients by SD-OCT and 6 x 6 mm OCT angiography scans, and both were found to be correlated with BCVA. SD-OCT along with OCTA could be a good technique for quantitatively evaluating different CSCR courses.

## Introduction

Central serous chorioretinopathy (CSCR) is a frequent condition that ranks fourth among pathological causes of vision loss in the retina, following age-related macular degeneration, diabetic retinopathy, and retinal vein occlusion, with an incidence of 1/10,000 [1-4]. Although most of them do not initially cause significant impairment to visual function, they do cause vision loss as well as symptoms such as central scotomas, distortion, reduced contrast sensitivity and color saturation, and so on. This is a chronic, recurring condition that creates several challenges in daily life and work, particularly in fields that require people to have high visual demands. Although no underlying pathophysiologic mechanisms have been established, CSCR is assumed to occur as a result of hyperpermeable choroidal capillaries, which, in conjunction with retinal pigment failure, creates a serous detachment of the neurosensory retina [5-7]. Acute CSCR is a self-limiting syndrome with neurosensory retinal detachment resolution and often good visual acuity (VA) recovery within three months [8]. Furthermore, chronic CSCR is defined as persistent subretinal fluid for more than six months [9].

Although CSCR can be diagnosed clinically, diagnostic tests such as fluorescein angiography (FA) and optical coherence tomography (OCT) are frequently used to rule out other possible illnesses and guide treatment [10]. Time domain OCT (TD-OCT), spectral domain OCT (SD-OCT), spectral domain OCT angiography (SD-OCTA), and swept source OCT (SS-OCT) are examples of OCT generations [11-13]. They are similar to ultrasound, but instead of sound waves, light is used. The main difference is that the velocity of light is much faster than the velocity of sound (3 x 108 m/s and 1500 m/s, respectively). Recent researches using structural OCT in B scan indicated a wide range of anatomic alterations between acute and chronic CSCR [14]. Nevertheless, changes in the retinal vascular microstructure between the two courses of CSCR were rarely reported. OCTA has recently made it possible to analyze the superficial and deep capillary plexus morphologically and quantitatively. This study aims to report the findings of retina microvasculature and choroidal changes using SD-OCT and OCTA, by assessing the superficial retinal capillary vessel density (SRVD) and subfoveal choroidal thickness (SFCT) quantitatively in acute and chronic CSCR patients compared to healthy subjects and to determine their correlations with visual acuity.

## Materials and methods

This cross-sectional, case-control study was approved by the Ho Chi Minh City Eye Hospital and Pham Ngoc Thach University of Medicine (IRB number 0522/PNT). All participants followed the principles of the Helsinki Declaration. Patients provided informed consent after being informed about the nature and potential effects of the study.

Based on the study by Mao et al., we computed the required sample size for the comparison of two independent means [15]. The result was 71 patients with CSCR in one eye, but because we were looking for higher precision, we surveyed more patients (91 in particular).

All study subjects were recruited from the Ho Chi Minh City Eye Hospital between May 2022 and October 2022. CSCR was diagnosed in all patients by one or more serous retinal detachments (SRDs) or pigmented epithelium detachments in the macular area detected by OCT. Patients with CSCR were divided into two separate groups: acute CSCR (onset of symptoms or SRD within three months) and chronic CSCR (onset of symptoms or SRD for more than six months) [9]. OCT was used to monitor the chronicity of CSCR on a monthly basis. Age-controlled participants with no history of systemic or ocular disease were enrolled. All patients' corticosteroid levels and cigarette/smoking habits were documented.

The exclusion criteria were as follows: (1) having different types of retinopathy or systemic diseases that could impact the retina, (2) CSCR in both eyes, (3) pregnancy, (4) other eye diseases that might have an impact on the retinal microvascular flow, (5) a high diopter of more than -6 dpt or a long eye axis of more than 26.0 mm, (6) OCTA image quality less than 7/10 and (7) refusing to participate in the study.

Slit lamp examination, intraocular pressure measurement, measurement of BCVA (reported as a logarithm of the minimum angle of resolution, or logMAR, equivalents), structural OCT (Zeiss Cirrus^TM^ 5000 HD-OCT; Carl Zeiss Meditec, Inc., CA), and OCTA (Cirrus^TM^ with AngioPlex^TM^; Carl Zeiss Meditec, Inc.) were all performed for all study subjects. The BCVA assessment, structural OCT, and OCTA were all performed on the same day. If both eyes of the same patient matched all of the inclusion criteria but none of the exclusion criteria, the eye with the lower VA was selected as the subject eye. The mean value of the subfoveal choroidal thickness on OCT was calculated by collecting data from both the horizontal and vertical directions. The SFCT was calculated by measuring the distance between the inner surface of Bruch's membrane and the innermost choroid-sclera interface.

All study participants underwent a 6 x 6 mm OCTA. The en face OCT was automatically separated into four layers using OCTA self-software (AngioPlex OCT angiography): superficial, deep, outer retina, and choroidal capillary. However, in this study, only OCTA variables of vessel density on the superficial layer were collected.

SPSS Statistics, version 20 (IBM Corp., Armonk, NY) was used for all statistical analyses. Data were reported as means ± standard deviation for statistical comparison. The Shapiro-Wilk test was used to check the normality of all counting variables prior to analysis: for quantitative variables with normal distribution, we employed Student's t-test; otherwise the Mann-Whitney U test was used. In multigroup comparisons, we utilized one-way analyses of variance. Pearson's correlation analyses were used to examine the relationships between continuous variables. A p value of less than 0.05 was considered significant.

## Results

Patient characteristics

The study included 91 patients (182 eyes), with 74 eyes in the unilateral acute CSCR group and 17 eyes in the unilateral chronic CSCR group; 91 eyes in the control group were CSCR patients' unaffected other eyes. There were 71 male and 20 female subjects (78% and 22%, respectively), with a male/female ratio of 3.55/1. The average age of the study participants was 40.78±1.26 years (range 23-59 years). The number of patients with unknown risk factors was 50, which amounted to 54.9%. The average BCVA was 0.36 ± 0.05 logMAR (range 0.20-0.40 logMAR). The prevalence of disease in the right and left eyes was comparable (45 and 46 patients, respectively). A detailed characterization of all patients is given in Table 1.

**Table 1:** Patient characteristics CSCR: central serous chorioretinopathy; BCVA: best-corrected visual acuity; logMAR: logarithm of the minimum angle of resolution

Patient characteristics	Frequency	Percentage
Age (years)		
<30	3	3.3
31–45	73	80.2
>45	15	16.5
Gender		
Male	71	78.0
Female	20	22.0
CSCR eye		
Right eye	45	49.4
Left eye	46	50.6
BCVA		
0–0.10 logMAR	16	17.6
0.20–0.40 logMAR	46	50.5
>0.40 logMAR	29	31.9

OCT analysis of the subfoveal choroidal thickness

On comparing baseline characteristics between CSCR eyes and normal eyes, CSCR eyes were found to have a thicker SFCT than normal ones (357.2 ± 11.8 μm and 290.4 ± 8.5 μm, respectively). In SFCT, there was a statistical difference (p < 0.00001) (Table 2).

**Table 2:** Baseline characteristics of CSCR eyes and normal eyes SFCT: subfoveal choroidal thickness; CSCR: central serous chorioretinopathy

	SFCT (μm)	Statistical value t	p value
CSCR eyes	357.2 ± 11.8	13.27	<0.001
Normal eyes	290.4 ± 8.5		

Decreased VD on superficial retina in acute CSCR patients

When compared to unaffected eyes, acute CSCR eyes revealed statistically significant increases in the central and inner area of superficial vessel density. There was no significant difference between chronic CSCR eyes and normal eyes (p > 0.05). The results of paired t-test variables when comparing chronic CSCR, acute CSCR, and controls are presented in Table 3.

**Table 3:** SRVD variables of chronic CSCR, acute CSCR, and controls SRVD: superficial retinal vessel density; CSCR: central serous chorioretinopathy

	SRVD			
	Whole retina	Fovea	Inner	Outer
Chronic (n = 17)	40.5 ± 3.4	24.2 ± 5.0	40.4 ± 4.0	41.2 ± 3.3
Acute (n = 74)	42.2 ± 1.5	28.0 ± 2.3	43.0 ± 1.4	42.5 ± 1.6
Controls (n = 91)	40.9 ±1.4	21.7 ± 2.0	39.9 ± 1.5	41.5 ± 1.3
p value between groups				
Chronic vs. controls	0.58	0.52	0.16	0.36
Acute vs. controls	0.60	<0.05	<0.05	0.72

Correlation between OCTA variables, structural OCT variables, and BCVA

**Figure 1:**
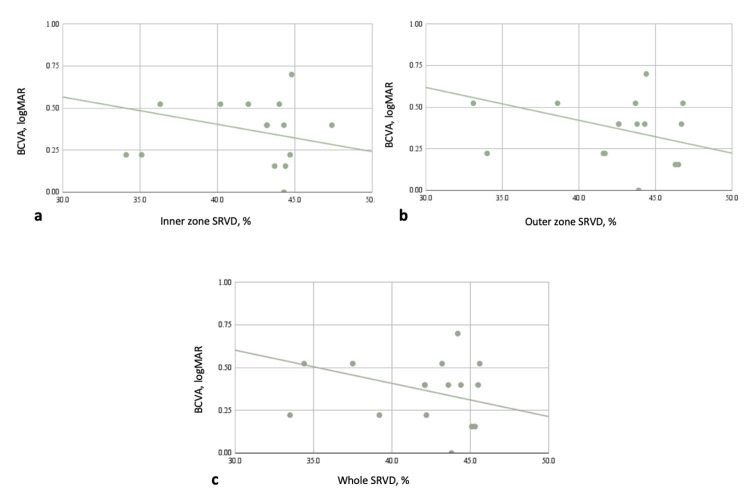
Correlation between OCTA variables and BCVA (logMAR) in chronic CSCR Negative correlations were found between the SRVD [inner zone (a), outer zone (b) and whole (c)] and BCVA. CSCR: central serous chorioretinopathy; OCTA: optical coherence tomography angiography; BCVA: best-corrected visual acuity; SRVD: superficial retinal vessel density; logMAR: logarithm of the minimum angle of resolution

**Figure 2:**
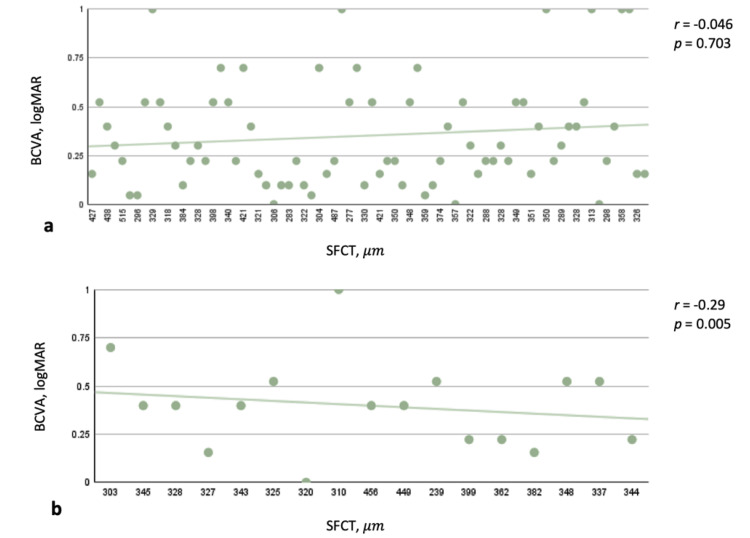
Correlation between SFCT (μm) and BCVA (logMAR) in acute and chronic CSCR (a) No correlation was found between the SFCT and BCVA in acute CSCR patients. (b) A negative correlation was indicated between the SFCT and BCVA in chronic CSCR patients. BCVA: best-corrected visual acuity; SFCT: subfoveal choroidal thickness; logMAR: logarithm of the minimum angle of resolution; CSCR: central serous chorioretinopathy

Table 4 shows that there was no statistical association between OCTA characteristics and BCVA in acute CSCR. However, in chronic CSCR, BCVA was significantly correlated with SRVD of the whole retina, particularly the inner and outer zones (all p < 0.05; Figure 1). Other OCTA variables were unrelated to BCVA (all p > 0.05). In terms of structural OCT variables, BCVA was correlated with SFCT in chronic CSCR (r = -0.29, p = 0.005; Figure 2).

**Table 4:** Correlation coefficient between OCTA variables, OCT variables, and BCVA CSCR: central serous chorioretinopathy; OCT: optical coherence tomography; SFCT: subfoveal choroidal thickness; OCTA: OCT angiography

	Acute CSCR (n = 74)		Chronic CSCR (n = 17)	
	r	p value	r	p value
OCT variables				
SFCT (μm)	-0.046	0.703	-0.29	0.005
OCTA variables				
Whole retina	-0.074	0.53	-0.581	0.014
Fovea	0.076	0.52	-0.268	0.298
Inner	0.001	0.99	-0.575	0.016
Outer	-0.098	0.41	-0.576	0.015

## Discussion

This was a cross-sectional, case-control study with 91 subjects (182 eyes). We quantitatively assessed and compared parameters in OCTA, structural OCT, and BCVA among acute CSCR, chronic CSCR, and healthy controls (patients’ unaffected fellow eyes). The major findings of this study are as follows: (1) the mean SFCT of CSCR eyes was greater compared to healthy controls, and the difference was statistically significant; (2) when compared to unaffected eyes, acute CSCR eyes showed statistically significant increases in the central fovea and inner zone of the superficial retinal vessel density. The difference between eyes with chronic CSCR compared to controls was not statistically significant; (3) an increased SFCT in acute CSCR correlated with lower VA; (4) when compared to unaffected eyes, acute CSCR eyes showed statistically significant increases in the central fovea and inner zone of the superficial retinal vessel density. The difference between eyes with chronic CSCR compared to controls was not statistically significant.

Central serous chorioretinopathy is a retinal disease characterized by serous retinal detachment with or without pigment epithelium detachment [2]. The alteration is primarily localized in the macular area, with fluid accumulating in the subretinal space. Several explanations concerning the disease's pathophysiology have been offered, two of which are accepted: choroidal dysfunction and pigment epithelial dysfunction [5,16]. Although the exact mechanism of the disease has not been determined, radiographic studies have revealed that the predominant alteration in the CSCR eye is due to increased permeability, which results in systemic fluid leakage from choroidal capillaries into the subretinal area [17]. The study's findings support the idea that an increase in choroidal capillary permeability will result in an increase in the thickness of the choroidal area located below the macula. When the choroidal capillaries become dysfunctional and have increased vascular permeability, the endothelial hydrostatic pressure increases, disrupting the connections of the pigment epithelial cells, causing the pigment epithelium to shed, and creating localized fluid accumulation underneath the retina. These anomalies will cause the choroid to thicken with time, notably the choroidal area below the macula. Maruko et al. also showed that in patients with disease in one eye, the choroidal thickness in the other eye was not thickened when compared to the diseased eye using the indocyanine green angiography (ICGA) imaging approach [18].

SRVD in the fovea and inner zone increased statistically significantly in acute CSCR eyes compared to normal eyes in an assessment of 91 patients (182 eyes), including 74 eyes with acute CSCR, 17 eyes with chronic CSCR, and 91 normal eyes. The difference between chronic CSCR eyes and normal eyes was not statistically significant. When Doppler ultrasound was performed on individuals with central serous chorioretinopathy, researchers discovered a decrease in blood flow in the posterior segment arteries, as well as a negative correlation between SFCT and arterial perfusion. This could be explained by the following hypothesis: when there is an accumulation of subretinal fluid in patients with central serous chorioretinopathy, it produces acute choroidal capillary dysfunction. Because of the blood supply disorder, the retina's immediate response is to increase blood supply to compensate, so in patients with acute CSCR, it is possible to see a statistically significant increase in the perfusion density of the superficial vascular system of the entire macula and the inner zone. Due to the disease's normal process, which is gradually regressing, the superficial layer perfusion density will also reduce, but not completely, as observed in the study results; therefore, these indexes will also increase. Yet, the difference is not statistically significant in chronic CSCR.

In our study, the result showed that both SRVD (whole retina and parafovea) and SFCT in chronic CSCR had a negative correlation with BCVA. Comparing to Mao’s study in 2020, chronic CSCR had a considerably lower vessel density on the superficial retina than acute CSCR and controls; additionally, there was no statistically significant difference between acute CSCR and controls. There was no correlation between the vessel density on the superficial retina and visual acuity in both chronic and acute CSCR [15]. Anatomically, superficial capillary plexus is located in the inner retina and primarily nourishes the inner layers [19]. The thickness of the choroidal layer underneath the macula in patients with central serous chorioretinopathy causes malfunction of the choroidal capillaries, which quickly leads to impaired perfusion of the outer retina, leading to a lack of oxygen and nutrients in this area. As a result, it affects photoreceptor function, resulting in vision loss, as demonstrated by the reported research findings. However, because this process takes a long time, vision will be unaffected if the disease is only in its acute phase and the lesions are still new. Furthermore, pseudo-presbyopia is a typical symptom in patients with central serous chorioretinopathy; since the subretinal fluid elevates the center of the macula, the focal point is shifted behind the retina, which causes the retina to wear down and adds to visual loss. In addition, in chronic CSCR, because this is the late stage, the SRVD parameters will not change much from the control eyes, but the actual changes in the eyes at this time are diminished nourishment and blood flow to the eyes. As a result, the retina causes a decrease in photoreceptors, resulting in a loss of vision. Hence, a negative correlation between SRVD variables, SFCT and BCVA was discovered.

There are some limitations to this study. We identified the start of the duration period as the first time the patient arrived at our hospital or based on their complaints. There were some differences between the two courses in terms of minor CSCR symptoms. Additionally, the distribution of patients in the groups was not similar and the parameters considered were insufficient; also, patients with underlying diseases or comorbidities were excluded from this study, as were cases with CSCR in both eyes. Considering all these factors, further studies should be done to address these shortcomings.

## Conclusions

In this study, we found that both altered SFCT and SRVD were identified in CSCR patients by SD-OCT and 6 x 6 mm OCT angiography scans, and both were found to be correlated with BCVA. SD-OCT along with OCTA could be a good technique for quantitatively evaluating different CSCR courses. Further research needs to be conducted to evaluate the choroidal thickness in the nasal and temporal regions.
